# Characterisation of sucking dynamics of breastfeeding preterm infants: a cross sectional study

**DOI:** 10.1186/s12884-017-1574-3

**Published:** 2017-11-17

**Authors:** Donna T. Geddes, Kok Chooi, Kathryn Nancarrow, Anna R. Hepworth, Hazel Gardner, Karen Simmer

**Affiliations:** 1 School of Molecular Sciences, Faculty of Science M310, The University of Western Australia, Perth, WA 6009 Australia; 2 Centre for Neonatal Research and Education, King Edward Memorial Hospital, Perth, WA Australia; 3 School of Paediatrics and Child Health, The University of Western Australia, Perth, WA Australia

**Keywords:** Breastfeeding, Preterm, Infant feeding, Lactation, Premature, Infant

## Abstract

**Background:**

Full breastfeeding is the ultimate aim for preterm infants to ensure they receive the full benefits of human milk however, preterm infants face a number of challenges associated with their immaturity and associated morbidities. In order to facilitate oral feeding, it is essential to have a sound knowledge of the sucking dynamics of the breastfed infant. The aim of this study was to measure and describe the sucking dynamics of the preterm breastfeeding infant.

**Methods:**

A prospective cross sectional observational study was carried out at King Edward Memorial Hospital, Perth. 38 mothers and their preterm infants (birth gestation age: 23.6–33.3 weeks; corrected gestation age 32.7 to 39.9 weeks) were recruited. Intra-oral vacuum levels, tongue movement and milk intake for a single breastfeed was measured. Statistical analysis employed linear regression and linear mixed effects models.

**Results:**

Synchronised ultrasound and intra-oral vacuum measurements show that the preterm infant generates vacuum by lowering their tongue in a parallel fashion, without distortion of the nipple/nipple shield. Baseline (B), mean (M) and (P) peak suck burst vacuums weakened over the course of a feed (B: *p* = 0.015; M: *p* = 0.018; P: *p* = 0.044) and mean and peak vacuums were weaker if the mother fed with a nipple shield (M: *p* = 0.012; P: *p* = 0.021). Infant milk intakes were higher when infants sucked for longer (*p* = 0.002), sucked for a greater proportion of the feed (p = 0.002), or had a greater sucking efficiency (*p* < 0.001).

**Conclusions:**

Breastfeeding preterm infants generated intra-oral vacuum in the same manner as term infants. Nipple shields were associated with weaker intra-oral vacuums. However, vacuum strengths were not associated with milk intake rather time spent actively sucking was related to milk volumes. Further research is required to elucidate factors that influence preterm infant milk intake during breastfeeding.

## Background

Breastfeeding provides advantages for both the mother and infant [1–5] that cannot be replicated by either artificial milks or artificial feeding methods [6]. Human milk itself, confers passive immunity to the infant, protecting against infection [7], which is highly advantageous to the immature preterm infant. Human milk also plays a role in the colonization of the preterm infants gut [8, 9], by providing beneficial bacteria [10], along with milk components such as human milk oligosaccharides (HMOs) that disrupt mechanisms leading to necrotizing enterocolitis (NEC) [11] through influencing bacterial growth [12]. Further, the incidence of NEC is six times higher in formula-fed infants compared to human milk fed infants [13, 14].

Whilst the ultimate nutritional goal is to achieve full breastfeeding in preterm infants, many variables influence the establishment and continuation of lactation, including nutritional [15, 16], biological [17, 18], psychological [19], cultural [20, 21] and social [22] components, all of which are vastly different for the mother/pre-term infant dyad compared to the mother/term infant dyad. Further, the establishment of breastfeeding is fraught with problems associated with the immaturity and health of the preterm infant [23]. Often the preterm infant is too ill to breastfeed immediately, and is tube-fed while oral feeding is established. During this time the initiation and establishment of a maternal milk supply is a priority [24]. Achieving and maintaining a plentiful milk supply is hampered by a tendency for the initiation of lactation to be delayed and less milk to be expressed compared to mothers of term infants [25, 26]. Recent evidence has suggested that expressing milk within an hour of delivery has a significant impact on milk volumes produced at 6 weeks post partum [27]. However, there is also a strong relationship between the frequency of pumping and milk volume [28, 29].

Indeed for breastfeeding to be successful, adequate amounts of milk must be removed frequently from the breast to ensure continued milk synthesis [30, 31]. While term infants receive 500 to 1000 mL/day [32], pumping studies show that milk production in the preterm mother tends to be maintained at around 340 to 640 mL/day [33, 34]. Similar to pumping studies [35], recent ultrasound studies of breastfeeding term infants, indicate that vacuum is instrumental in milk removal [36, 37] and this is commensurate with preterm bottle-fed infants, where it has been shown that the development of vacuum strength and rhythmicity over time is necessary to improve the feed effectiveness and efficiency [38, 39]. Furthermore, adequate coordination of sucking, swallowing and breathing [40–43] is essential for safe feeding and due to neurological and developmental immaturity; coordination of sucking, swallowing and breathing may be compromised and then further compounded by respiratory conditions.

Few objective studies have been carried out to investigate the mechanisms of breastfeeding in the preterm infant, often presumed to be different or ‘immature’ compared to term infants. This belief has been based on clinical observation and measurement of intra-oral vacuums, but no studies have imaged the sucking mechanism of breastfed preterm infants yet this is essential, to devise successful strategies focused on facilitating full breastfeeding. Recent ultrasound studies of term breastfeeding infants confirm that term breastfeed infants employ a parallel movement of the anterior and mid tongue to remove milk from the breast and use a more wave like motion of the posterior tongue to clear the milk bolus from the oral cavity during the oral phase of swallowing [44–46].

## Methods

The aims of this study were to measure and describe the sucking dynamics of the preterm breastfeeding infant feeding with a nipple shield (tongue movement and intra-oral vacuum) and explore relationships with milk intake.

### Participants

A convenience sample of 47 mothers and infants (birth gestation age: 23.6–33.3 weeks; corrected gestation age (CGA) 32.7 to 39.9 weeks) was recruited from the special care nurseries of King Edward Memorial Hospital for Women (KEMH), Perth between 1 August 2011 and 30 June 2012. Five infants were discharged before a feed could be monitored and 4 were excluded due to technical issues recording intra-oral vacuum and ultrasound images, leaving a sample size of 38. These mothers were a subset of a larger cohort taking part in a randomized controlled trial to assess the efficacy of a novel feeding system (AustralianNewZealandClinicalTrialsRegistry, ACTRN12614000875606, http://www.ANZCTR.org.au/ACTRN12614000875606.aspx). The infants recruited to the study were healthy preterm infants admitted to the NICU, whose mothers intended to breastfeed. Nipple shields are used to facilitate the establishment of breastfeeding particularly in infants having difficulty maintaining attachment to the breast. Exclusion criteria included oro-facial anomalies that might affect feeding, as well as intraventricular hemorrhage and other congenital anomalies. All infants were required to be successfully latching and sucking at the breast before participating in the study. Mothers supplied written, informed consent to participate in the study, which was approved by the Scientific Research Ethics Committee of King Edward Memorial Hospital. One breastfeed was monitored for each infant.

### Infant milk intake

Milk intake was determined by test weights taken immediately before and after the breastfeed [32] (Baby Weigh Scale, Medela AG, Baar, Switzerland). The difference in weight (g) was equivalent to the transfer volume (mL). Milk transfer rate (mL/min) was calculated by dividing the volume of milk (mL) consumed by the duration of the feed (minutes). Intra-oral vacuum was measured using the methods previously described by Geddes et al., 2008 [36].

### Ultrasound imaging

Submental ultrasound scans of the midline of the infant’s oral cavity were acquired [47–49] using a portable TITAN ultrasound system, with an endocavity convex transducer ICT 8–5 MHz (SonoSite Inc.). Details regarding this technique have been described in detail previously [36]. Signals from the ultrasound machine and pressure transducer were recorded simultaneously with a Video Capture Module (ADInstruments). This module allows synchronized recording and playback of a movie file and LabChart data, allowing analysis of the tongue movement in relation to the vacuum cycle. Recording of the data was begun prior to the infant latching to the breast and ceased at the end of the feed.

The first three well-visualized nutritive suck cycles were selected from each feed. Tongue and nipple movement were measured on two still images from each suck cycle when the mid tongue was at its highest (TU) and lowest points (TD) using Screen Calipers v 3.2 (Iconico Inc.). Measurements made were: nipple to hard–soft palate junction (N-HSPJ), intra-oral depth (IOD; vertical measurement of the mid tongue lowering creating the space accommodating the milk bolus), and nipple diameter at 2, 5, 10, 15 and 20 mm from the tip of the nipple.

### Measurement of infant intra-oral pressure

Infant intraoral pressures were measured via a small silastic tube filled with sterile water and taped alongside the nipple and attached to a disposable pressure transducer (Cobe Laboratories, Frenchs Forest, NSW, Australia). The transducer was connected to an amp bridge (ADInstruments, Castle Hill, NSW, Australia) and the output was recorded using MacLab (ADInstruments) and software package Chart v5.0.2 (ADInstruments) on a laptop computer (Mac OS X v10.3.8).

Suck burst measurements made were: mean minimum pressure (peak vacuum) and mean maximum pressure (baseline vacuum), mean pressure, suck rate and duration of the suck burst. Mean pressure and pause duration were measured for the pauses between the suck bursts.

### Statistical analysis

Analyses were performed using R 3.0.3 for Mac OS X [50]. Packages nlme [51] and lattice [52] were used for linear mixed effects models and graphical exploration, respectively. Differences were considered significant when *p* < 0.05. Descriptive statistics are presented as mean ± sd (range) and/or median [IQR] otherwise. Parameter estimates are presented as estimate [95% confidence interval (CI)] and included where relevant due to transformation of some of the variables.

Welch two sample *t*-tests were used to test for differences between infants who had and had not achieved full suck feeds and between infants fed with and without a nipple shield. Linear regression was used to test for univariate associations between milk intake (square root transform) and feed characteristics (prescribed volume, average vacuum across the feed, feed duration, time pausing, number of suck bursts, number of single sucks, percentage of single sucks).

All other variables: NHSPJ, IOD, nipple diameter, measurement location (nipple diameter), time elapsed since beginning of feed; mean, baseline and peak vacuums; burst duration; sucking rate; use of nipple shield during the feed, current weight, CGA, postnatal age, birth gestational age and weight, age at introduction of suck feeds and achievement of full suck feeds, and the timing of the monitored feeds were analysed using linear mixed effects models to account for the related nature of the data. Data was grouped by infant, with alternate grouping of tongue position within infant considered for all tongue movement variables. Significant effects of tongue position were considered to indicate individual differences in degree of movement. A simultaneous linear regression model approach was used to investigate frequency of significant patterns within infants and whether these patterns were consistent.

Tongue movement, vacuum, and sucking patterns data were analysed as with linear mixed effects models. Random effects considered were random intercepts, and in models with time based variables, individual time patterns. Random effects were only included if the term was also included in the fixed effects. Suitability of random effects was tested using likelihood ratio tests. Random effects other than random intercepts were considered to indicate different patterns between groupings.

Analysis specific fixed effects related to the research question included measures that changed for example when the tongue was either up or down and included tongue position (NHSPJ, IOD, nipple diameter), measurement location (nipple diameter), time elapsed since beginning of feed, either as linear or 2nd order polynomial (mean, baseline and peak vacuums; burst duration; sucking rate). Covariates of interest that were considered to potentially affect the relationship/s between the response variable/s and the fixed effects were use of nipple shield during the feed, current weight, CGA, postnatal age, birth gestational age and weight, age at introduction of suck feeds and achievement of full suck feeds, and the timing of the monitored feeds relative to these last two. Final models were selected using stepwise modeling, omitting covariates with marginal significance <0.05, starting with analysis specific fixed effects. Fixed effects were tested by considering the set of models that included one additional covariate, interaction, or higher order term; this was considered to be the final model when only terms that were considered to be significant as fixed or random effects were retained. Appropriateness of model fits were assessed visually from standard residual plots.

Sucking rate was analysed as per vacuum variables. Measurements of >200 sucks per minute were omitted (*n* = 9, leaving *n* = 1681 records for analysis) as these are artifacts of the measurement process. Number of sucks per burst was grouped as ‘single’ (1 suck), <10 sucks and 10+ sucks. Linear mixed effects models with absolute max or absolute min vacuum as response and suck burst category as the fixed effect were used to assess how vacuum and number of sucks related; Tukey’s all pair comparisons was used to determine which suck burst categories were different. No covariates were considered.

## Results

### Participants

Infant (22 female, 16 male) characteristics are shown in Table 1. Missing data for the 38 infants analysed include: weight at the study session (*n* = 1), milk intake and sucking efficiency (*n* = 3); tongue movement data could not be measured due to technical errors in video recording for 9 infants.

**Table 1:** Infant characteristics of preterm infants monitored for a single breastfeed

	Mean ± SD	Median [IQR]	Range
Birth			
age (weeks)	29.9 ± 2.8	30.6 [28.1, 32.1]	23.6–33.6
weight (g)	1364 ± 432	1358 [1085, 1716]	540–2080
Current			
age (CGA, weeks)	35.8 ± 1.6		32.7–39.9
age (post natal, weeks)	5.9 ± 3.8	5.2 [3.4, 7.4]	1.0–16.3
weight (g)	2118 ± 385		1430–3220
Suck feeds			
Introduced (CGA, weeks)	33.4 ± 0.8	33.3 [32.9, 33.9]	31.4–35.3
Introduced (post natal, weeks)	3.4 ± 2.7	2.8 [1.3, 4.9]	0.4–10.6
Full sucks (CGA, weeks)	36.8 ± 1.2	36.7 [36.0, 37.3]	34.9–40.1
Full sucks (post natal, weeks)	6.9 ± 3.6	6.0 [4.2, 8.7]	2.4–16.6
Study day			
weeks since first suck	2.4 ± 1.6	2.1 [1.2, 3.7]	0–5.7
weeks since full sucks^a^	−1.0 ± 1.2	−1.0 [−1.7, −0.3]	−3.9-1.1

^a^Weeks since full sucks: negative values indicate the infant was monitored x weeks prior to full suck feeds achieved

Eight infants had achieved full suck feeds and all but 6 infants were fed using a nipple shield. Infants that had reached full suck feeds were older (CGA; *p* = 0.003) and use of a nipple shield was not associated with CGA (*p* = 0.34).

### Feed characteristics

Feed characteristics are detailed in Table 2. Three infants had no measurable milk intake (0 mL) and 7 had milk intakes of 4 mL or less. Milk intakes were higher when infants sucked for a longer time (*p* = 0.002), spent a greater proportion of the feed sucking (*p* = 0.002), or had a greater sucking efficiency (*p* < 0.001). Effect sizes were small (estimate [95% CI]: additional 0.07 [0.01–0.18] mL for each additional minute sucking; 0.06 [0.01, 0.16] mL for each additional 5% of feed spent sucking; 0.07 [0.02, 0.15] mL for each additional ml/min efficiency) reflecting the typically small milk intakes and the large variation in sucking durations, feed proportions, and efficiency. No associations were seen with other considered covariates.

**Table 2:** Feed characteristics of infants during a monitored breastfeed (ultrasound imaging and measurement of intra-oral pressure)

	Mean ± SD	Median [IQR]	Range
Feed duration (min)	13.8 ± 7.3	11.4 (8.3, 18.1)	2.4–28.6
Sucking duration (min)	5.0 ± 3.6	3.3 (2.2, 7.4)	1.6–11.9
Proportion (%) of feed sucking	38 ± 18	34 (24, 49)	10–86
Suck bursts			
Total	50.7 ± 34.7	39 (28, 64)	6–133
single sucks	6.2 ± 4.7	5 (3, 9.5)	0–18
% single sucks	13 ± 10	11 (6, 17)	0–38
Mean Vacuum (mmHg)	−40.6 ± 27.8	−33.0 (−46.6, −22.4)	−126.4 - -0.4
Milk intake (mL)	14.4 ± 13.4	12 (4, 21)	0–60
Prescribed volumes (mL)	45 ± 8	45 (40,50)	30–60
Sucking efficiency (mL/min)^*^	3.7 ± 4.1	2.3 (1.2, 4.4)	0–19.8

^*^ *n* = 35

### Characteristics of tongue movement

**Fig. 1:**
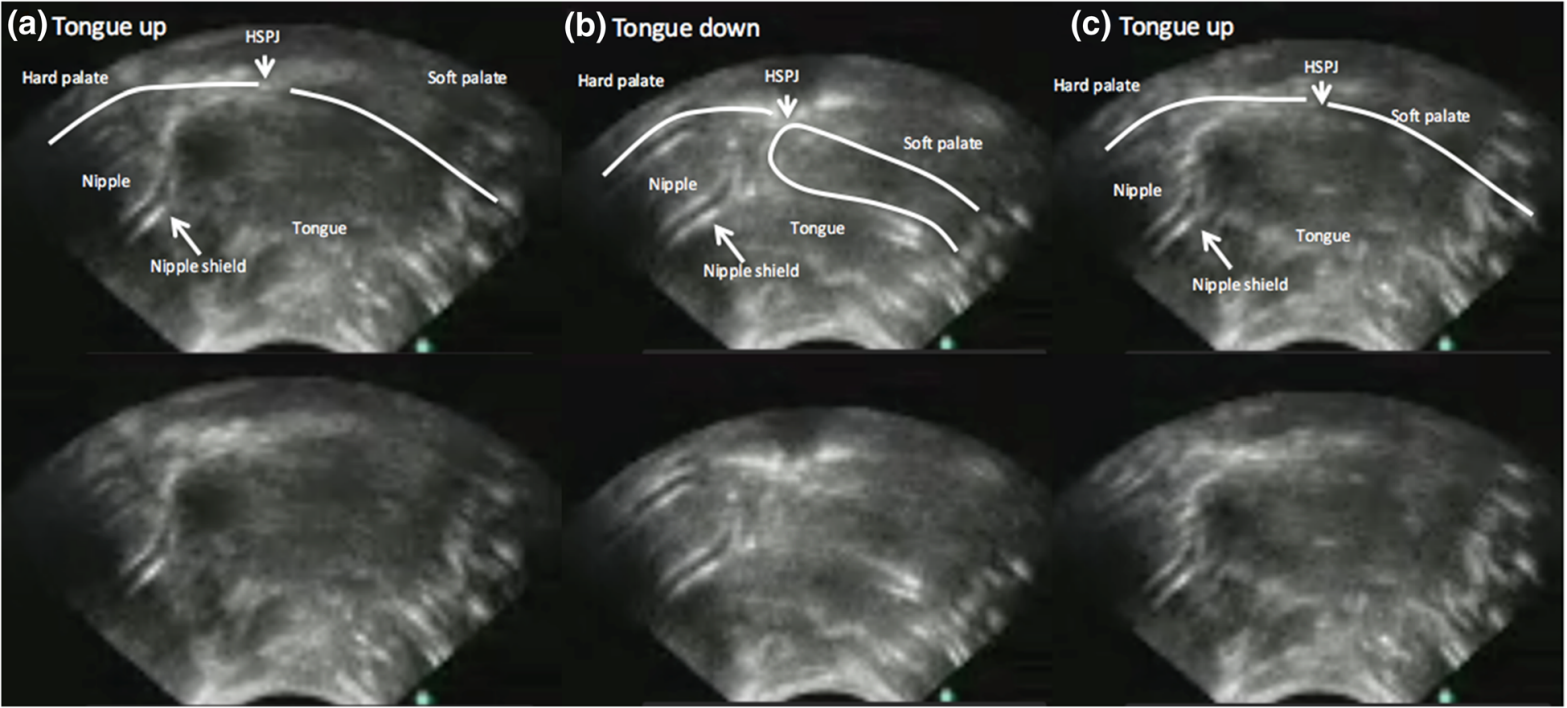
Ultrasound images of one cycle of a preterm breastfeeding infant feeding with a nipple shield (**a**) tongue up corresponds with baseline vacuum (**b**) when the tongue is lowered to the lowest point peak vacuum is applied to the breast and milk flows (**c**) tongue returns to the soft palate and milk is removed from the oral cavity

**Fig. 2:**
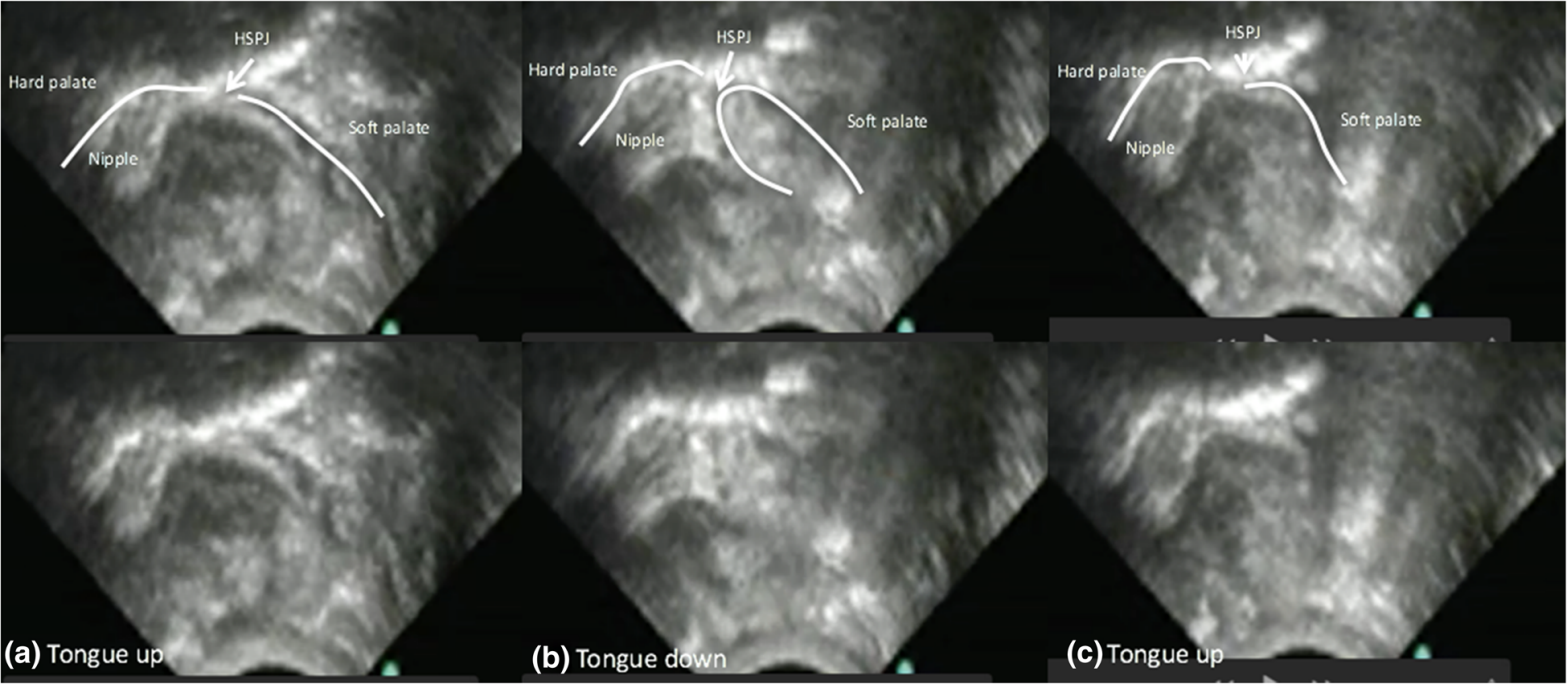
Ultrasound images of one cycle of a preterm breastfeeding infant (**a**) tongue up corresponds with baseline vacuum (**b**) when the tongue is lowered to the lowest point peak vacuum is applied to the breast and milk flows (**c**) tongue returns to the soft palate and milk is removed from the oral cavity

**Fig. 3:**
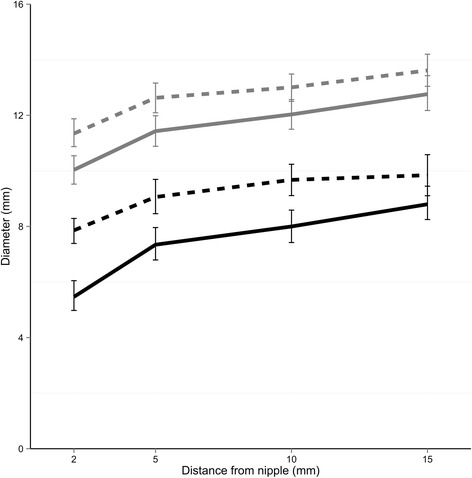
Nipple diameters plotted at different positions of the nipple (2, 5, 10 and 15 mm) for the breastfeeds with a nipple shield (grey lines; *n* = 32) and with**out** a shield black lines; *n* = 6). Continuous lines represent nipple diameter when the tongue is up and dotted lines when the tongue is down

Synchronised ultrasound and intra-oral vacuum measurements show, that as the infant tongue is lowered a vacuum (decreased negative pressure) is generated and the as the tongue is raised the vacuum decreases in strength (increased negative pressure) (Figs. 1 and 2). Ultrasound measurements of N-HSPJ, intra-oral depth and nipple diameters are given in Table 3. There were baseline differences between infants on all measures (*p* < 0.001 all cases). The average difference between tongue up and tongue down differs between individuals for intra-oral depth (IOD; 1.9–8.8 mm; *p* < 0.001) and nipple diameters (1.9–8.8 mm; 2 mm: −0.8, 4.2; 5 mm: −0.7 - 3.9; 10: −1.2 - 3.1; 15: −0.5 - 2.; p < 0.001; Fig. 3) but not for N-HSPJ distance (*p* = 0.47). For all infants IOD was significantly different for tongue up and tongue down (*p* < 0.003). Tongue up measurements are expected to be zero (tongue in apposition with the palate), 3 infants had average measures significantly different from zero (*p* < 0.026). For nipple diameters 3 infants showed different amounts of movement at different locations and there were 7 infants with no significant difference and no evidence of association with nipple shield use (*p* = 0.61).

**Table 3:** Tongue movement measurements at tongue up and tongue down (mean ± SD), for all infants and suck cycles. Nipple diameters are measured at 2, 5, 10, and 15 mm from the tip of the nipple and are separated by whether the feed was given with or without a nipple shield. *Nipple diameters are missing at tongue up for one infant; 15 mm location was not measurable on 12 tongue down and 14 tongue up images

	With nipple shield (*n* = 67^a^)		Without nipple shield (*n* = 18)		Combined data	
	Tongue up	Tongue down	Tongue up	Tongue down	Tongue up	Tongue down
N-HSPJ distance (mm)	7.0 ± 2.5	5.3 ± 2.2	4.8 ± 1.2	2.7 ± 1.0	6.5 ± 2.5	4.7 ± 2.3
Intra-oral depth (mm)	0.3 ± 0.5	4.3 ± 1.9	0.2 ± 0.6	3.4 ± 1.2	0.3 ± 0.5^*^	4.1 ± 1.8
Nipple diameters (mm)						
2 mm	10.0 ± 2.2	11.3 ± 2.1	5.5 ± 1.2	7.9 ± 1.0	9.1 ± 2.8	10.6 ± 2.4
5 mm	11.4 ± 2.2	12.6 ± 2.1	7.3 ± 1.3	9.1 ± 1.4	10.6 ± 2.7	11.9 ± 2.5
10 mm	12.0 ± 2.3	13.0 ± 2.1	8.0 ± 1.4	9.7 ± 1.3	11.2 ± 2.7	12.3 ± 2.4
15 mm	12.8 ± 2.2	13.6 ± 2.1	8.8 ± 1.1	9.8 ± 1.4	12.0 ± 2.6	12.9 ± 2.5

^a^IOD at tongue up is highly skewed: median [IQR] = 0 [0, 0.5]

When a nipple shield was used, N-HSPJ distances were on average 2.4 mm [95% CI: 0.5, 4.3] longer (*p* = 0.017), and nipple diameters were on average 3.9 mm (95% CI: 2.2, 5.6) larger than without a nipple shield. There was no association between nipple shield use and IOD (*p* = 0.21; mean: 0.5 mm; 95% CI: -0.3, 1.3). No significant interactions were seen between shield use and either tongue up or tongue down (N-HSPJ, *p* = 0.28; nipple diameters, *p* = 0.074) or location (nipple diameters, *p* = 0.11).

There were no significant associations with any of the other considered covariates, after accounting for tongue position, use of nipple shield, and in the case of nipple diameters, location of measurement.

### Intra-oral vacuum

Suck bursts were shorter than pauses (*p* < 0.001). Pause durations were univariately associated with age relative to full suck feeds (*p* = 0.019), but this association did not remain if any one of birth weight, postnatal age, age at introduction of suck feeds, or nipple shield use was accounted for. Suck burst durations were not associated with any of the considered covariates (*p* > 0.19).

**Fig. 4:**
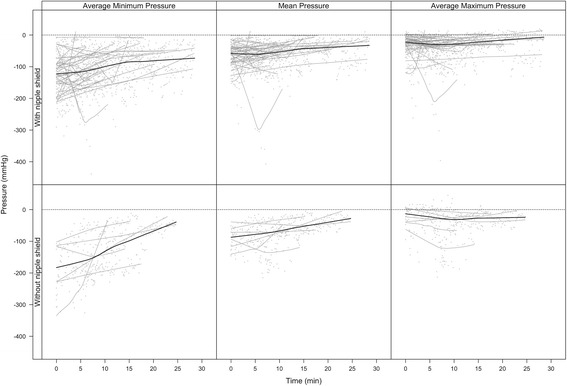
Average minimum (peak vacuum), mean and average maximum (baseline vacuum) pressures across a feed for infants that were breastfeeding with and without a shield

The variability of baseline vacuum and mean vacuum do not differ significantly (*P* = 0.062). Peak vacuum is significantly (*p* < 0.001) more variable than both baseline and mean vacuum (Fig. 4). Mean suck burst vacuums weakened over the course of a feed (Fig. 1; *p* = 0.018), and were weaker with a nipple shield (Fig. 1; *p* = 0.012) or if the infant had not achieved full suck feeds (*p* = 0.043). Pause vacuums also weakened over the course of a feed (*p* < 0.001).

**Fig. 5:**
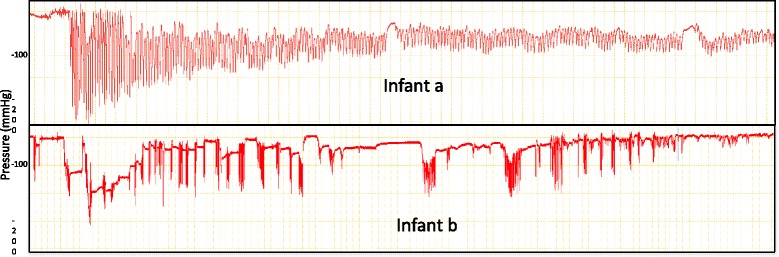
Intra-oral vacuum traces of a breastfeed for 2 preterm infants (**a**) this infant displays peak vacuums between −90 mmHg and – 250 mmHg and is able to maintain suck bursts with more than 10 sucks per burst (**b**) this infant applies peak vacuum between −10 mmHg and −150 mmHg and often has less than 10 sucks per suck burst and longer pauses than infant (**a**)

Baseline suck burst vacuums weakened across the course of a feed (Fig. 4; *p* = 0.015) with no other associations seen (*p* > 0.17). Peak suck burst vacuums also weakened across the course of the feed (Fig. 4 **;** *p* = 0.044), and were weaker when the infant was fed using a nipple shield (*p* = 0.021), and in infants who had not achieved full suck feeds (*p* = 0.004). For all measures, there were significantly different patterns between infants (Fig. 5; *p* < 0.001). No robust associations were seen with considered covariates including CGA, current weight and shield use. Further, the variability of baseline (*p* = 0.73), mean (*p* = 0.45) and peak (*p* = 0.38) vacuums were not significantly associated with milk intake.

Mean suck rates were 89 ± 7 sucks/min did not differ over the course of a feed (*p* = 0.47). Higher numbers of sucks per burst were associated with older infants (CGA; *p* = 0.008) and lower sucking rates (p < 0.001) in that for each additional suck, the sucking rate is on average 0.6 s (95% CI: 0.3, 0.9) slower. No overall associations were seen between sucking rate and baseline (*p* = 0.13) or peak (*p* = 0.09) vacuums, or with other considered covariates (*p* = 0.36).

Significant individual differences are seen for the associations between sucking rate and number of sucks in the burst (p < 0.001), the baseline and peak vacuums (p < 0.001, both), and changes in sucking rate over the course of a feed (p < 0.001).

Suck bursts of >10 sucks were present in 36/38 of feeds. This pattern was more common than single sucks, with 9 infants having over a third of suck bursts consisting of more than 10 sucks. Median [IQR] frequency of bursts >10 sucks was 8.5 [0, 45] and as a percentage of the number of suck bursts in the feed, on average 25% ± 18%. The median [IQR] number of single sucks was 5 [3, 9.5]. The proportion single sucks bursts ranged from 0 to 38% with a median [IQR] of 11% [6.25%, 17.5%].

## Discussion

Preterm infants are at risk of feeding difficulties due to the immaturity of their neurological and motor systems, which are magnified in those with underlying complications [53]. As delay in attainment of independent oral feeding may extend the time to discharge [54], a detailed and comprehensive knowledge of the physiology of feeding is essential to develop evidence-base guidelines and interventions that support the feeding maturation of the preterm infant and minimise long term behavioural eating disorders such as oral aversion [55, 56]. In this study we found that stable preterm infants removed milk from the breast by lowering their tongue in a manner similar to the tongue action of term breastfeeding infants however the vacuum created by the preterm infant is weaker than the term infant. The only factors related to effectiveness of milk removal were; duration of the feed and the time spent actively sucking at the breast.

It is often presumed that the preterm infant lacks the ability to suck or has an inefficient suck [57], however we have shown that in preterm infants with no major complications employ a sucking mechanism during breastfeeding that is similar to that of the term infant (Table 3; Figs. 1 and 2). That is the preterm infant lowered its tongue away from the palate to generate vacuum, reaching a peak vacuum when the tongue was at its lowest point. During the downward motion of the tongue the soft palate moved downwards and remained in contact with the posterior tongue. Milk flowed into the cavity bounded by the tip of the nipple/nipple shield, hard palate, soft palate and ventral surface of the tongue. As the tongue moved upwards the vacuum reduced in strength and the milk was cleared from the oral cavity. No marked peristaltic action of the tongue was evident (Fig. 1). This suggests a mature sucking motion has developed in utero, consistent with reports of thumb sucking identified by real time ultrasound imaging in utero as early as 15–21 weeks [58]. Therefore, ineffective and inefficient feeding performance in preterm infants is likely to be due the inability to generate adequate vacuum strength and/or disorganized sucking due to poor suck-swallow-breathe co-ordination.

Optimal positioning of the nipple in relation to the hard-soft palate junction should be such that the infant is able to remove an appropriate volume of milk, that can be cleared to the pharyngeal area during the oral phase of swallowing [57]. Interestingly, positioning of the nipple was not different whether a nipple shield was used or not (Table 3). And the N-HSPJ distance was comparable to that of term infants (TU: 6.5 mm; TD 4.7 mm) [45] despite the oral cavity of preterm infants being much smaller, indicating the existence of a possible ‘sweet spot’ where a bolus of milk can be accommodated, without triggering the gag reflex [59]. One might speculate that different sizes of shields could influence the positioning in the oral cavity, such that if it is too close to the HSPJ it may only allow a small bolus to be removed or may discouraging sucking. Alternatively, if the shield and nipple were located too distant from the HSPJ, the strength of vacuum applied might be reduced; resulting in a reduction in the amount of milk removed.

Movement of the nipple in the preterm oral cavity was similar to term infants overall (term: approx. 1.8 mm [45]; preterm: approx. 2.0 mm; Table 3; Fig. 1) and the downward movement of the mid tongue was highly variable between infants (IOD: 1.9 to 8.8 mm) and across the breastfeed (Fig. 3). Surprisingly, the average degree of IOD (Table 3) is similar to that of the term breastfeeding infant. Normally the tongue rests on the palate in the tongue up position, in term infants [45] however in 3 infants that was not the case. Two of the infants were fed with a shield, raising the possibility that the shield is too large and is restricting upward movement of the tongue. This would be similar to the issues encountered with women with large nipples where position of the nipple and tongue movement is likely impacted [60].

Nipple shields are often used to facilitate breastfeeding in preterm infants by enabling attachment to the breast and facilitating milk removal [61] however, there are conflicting reports as to whether they impact long term breastfeeding outcomes. A recent large cohort study found no relationship between nipple shield use and age at exclusive breastfeeding in one analysis [62] and an increased risk of not achieving exclusive breastfeeding (49% exclusively feeding with nipple shield use and 66% without) in another analysis [63]. It is not clear whether all factors known to influence exclusive breastfeeding were accounted for and frequency of use was not reported. A within infant study has shown that infants receive more milk from the breast when using nipple shields [61] therefore there is a clear need to understand how nipple shields function in the preterm infant. Better attachment to the breast was supported by the observation that baseline vacuum values of infants that fed with a shield were similar to the infants that were capable of feeding without a shield (Fig. 4). However, whilst we found that the preterm infants’ pattern of tongue movement during breastfeeding is similar to term infants and is not altered with nipple shield use they generated a much weaker intra-oral vacuum (mean vacuum: −40.6 ± 27.8) than term infants than term infants (−114 ± 50 mmHg). Since intra-oral vacuum is instrumental in milk removal from the breast and weak vacuum is associated with both poor milk intake and bottle-feeding efficiency [64] further research should focus on improving milk transfer rates during breastfeeding both with and without nipple shields. Factors that might influence milk transfer include the size of the nipple shield relative to the size of the nipple and infant’s oral cavity and the volume of milk in the breast (or degree of breast fullness) as this influences milk flow rates in that milk flow rates are faster with greater degrees of fullness [65].

The delay that preterm infants experience in the attainment of feeding milestones has been partly attributed to the infant’s inability to generate sufficient vacuum, as well as uncoordinated sucking, thereby reducing feed efficacy [64]. These factors are partly reflected in the large variability within and between infants, particularly across a breastfeed (Figs. 4 and 5). Most notably features differed to term infants; all suck burst vacuums (mean, baseline and peak) and pause vacuums reduced, across a breastfeed whereas baseline and pause vacuums strengthen across a breastfeed in term infants [66]. The reduction in vacuums across a feed is commensurate with clinical observations of the preterm infant tiring during the feed, relaxing its attachment to the breast/shield and becoming less effective at milk removal [61, 67].

Logically clinicians assume that as the infant grows and matures their ability to feed or create adequate feeding vacuums improves [64]. We did not find strength of vacuum to be related to either infant weight (birth-weight, weight at feed) or age (gestational age, corrected gestational age) casting doubt on the notion that larger, older infants are mature enough to create strong intra-oral vacuums compared to their younger, lighter counterparts [43]. These findings lend support to the practice of beginning feeding as soon as the infant achieves cardiorespiratory stability [68]. Certainly, according to Lau’s bottle-feeding data, one would expect older infants to progress more rapidly, however lower vacuums are typically sufficient to remove milk from the bottle compared to the breast [39]. The inability to create and maintain a substantial level of vacuum during breastfeeding to remove adequate volumes of milk could result from issues other than immaturity such as respiratory issues [69], poor suck-swallow-breathe coordination [70], nipple shields and the behavioral state of the infant during the breastfeed. Indeed, further investigation of the influence of these factors on the success of breastfeeding is warranted particularly across time.

Sucking rates of the preterm infants (89 ± 7 sucks/min) were similar to those documented for term breastfeeding infants [66, 71–73]. However, sucking rate was calculated excluding single sucks, which rarely occurs in term breastfeeding infants and may be another marker of maturation of infant feeding. Sucking rates remained relatively consistent over the course of the feed, whereas they increased across the breastfeed in term infants [66] and is speculated to be due to reduced milk flow rate, decreased availability of milk and infant satiety [66]. The lack of changes in the sucking rate in the preterm breastfeeding infant suggests an immature response to the breast, in that it may be difficult for the infant to suck consistently when milk is available during the milk ejection period [74]. Alternatively, low milk production and low volumes in the breast may reduce milk flow rate, thereby influencing preterm sucking patterns. It may also reflect an aspect of feed maturation, in that older infants tended to generate more sucks and suck more slowly during a breastfeed than the younger infants, however this was not associated with either baseline (latch) or peak vacuum suggesting that coordination of the suck swallow breathe process could also be impacting sucking rates.

Another suggested marker of developmental progress, based on observations of bottle-feeding, is the ability of infants to maintain 10 or more sucks for a suck burst, which reflects organized breathing [75, 76]. In this study, all but 2 infants (5%) exhibited at least one suck burst of 10 or more sucks, with on average only a quarter of the feed consisting of suck bursts >10 sucks. As mentioned previously, single sucks are rare in term breastfeeding infants compared to preterm infants and in this study single suck ‘bursts’ accounted for 11% of the feed (median; range: 0 to 38%). Potentially a reduction of single sucks during breastfeeding could be indicative feeding progression. Further studies monitoring preterm breastfeeding characteristics over time are required to elucidate changes in vacuum patterns.

The volumes of milk removed from the breast by the preterm infants in this study were low and, in some cases much lower than the amount prescribed to ensure adequate growth. For example, 26% of the infants received no milk (3/38) or <5 mL (7/38) and only one infant reached the prescribed volume. Whilst it is expected that stronger intra-oral vacuums during breastfeeding would result in increased milk intake, as demonstrated by Lau et al. [64] in preterm bottle-fed infants, we did not find this. Neither did we find a relationship between variability of vacuum strength and milk intake This maybe because preterm infants are required to suck more strongly at the breast and therefore fatigue more rapidly [67]. Thus improving milk transfer from the breast might require more emphasis on the breast and time spent actively sucking at the breast although the effect is small (average 0.07 mL/min spent sucking).

## Conclusion

Breastfeeding preterm infants generated intra-oral vacuum in the same manner as term infants albeit at a lower level. Intra-oral vacuum strengths were not associated with milk intake, rather the duration of the feed and the time spent actively sucking was related to milk volumes. Further research is required to elucidate factors that influence preterm infant milk intake during breastfeeding as well longitudinal studies to track maturation of breastfeeding skills.

## Abbreviations

B: Baseline
CGA: Corrected gestation age
HMO: Human milk oligosaccharide
IOD: Intra-oral depth
M: Mean
NEC: Necrotizing enterocolitis
N-HSPJ: Nipple hard-soft palate junction
P: Peak
TD: Tongue down
TU: Tongue up
